# Exposure to a biopesticide interferes with sucrose responsiveness and learning in honey bees

**DOI:** 10.1038/s41598-020-76852-2

**Published:** 2020-11-16

**Authors:** Daniele Carlesso, Stefania Smargiassi, Lara Sassoli, Federico Cappa, Rita Cervo, David Baracchi

**Affiliations:** 1 Department of Biology, University of Florence, Via Madonna del Piano, 6, 50019 Sesto Fiorentino, Italy; 2 Department of Biological Sciences, Macquarie University, Sydney, NSW 2109 Australia; 3 Department of Life Sciences and Systems Biology, University of Turin, Via Accademia Albertina 13, 10123 Turin, Italy

**Keywords:** Ecology, Microbiology, Neuroscience, Psychology, Zoology

## Abstract

The entomopathogenic fungus *Beauveria* *bassiana* is a widely used biopesticide that is considered as an effective alternative to classical agrochemicals*. B. bassiana* is thought to be safe for pollinators although little is known about its side-effects on pollinators’ behaviour and cognition. Here, we focused on honey bees and used the proboscis extension response (PER) protocol to assess whether *B. bassiana* affects individual sucrose responsiveness, non-associative and associative olfactory learning and memory. Fungus-treated bees displayed an enhanced sucrose responsiveness, which could not be explained by metabolic alterations. Strikingly, exposed bees were twice as inconsistent as controls in response to sucrose, showing PER to lower but not to higher sucrose concentrations. Exposed bees habituated less to sucrose and had a better acquisition performance in the conditioning phase than controls. Further, neither mid- nor long-term memory were affected by the fungus. As sucrose responsiveness is the main determinant of division of foraging labour, these changes might unsettle the numerical ratio between the sub-castes of foragers leading to suboptimal foraging. Although the use of biocontrol strategies should be preferred over chemical pesticides, careful assessment of their side-effects is crucial before claiming that they are safe for pollinators.

## Introduction

The honey bee *Apis mellifera* is considered one of the most valuable pollinators worldwide^1^. In the last few decades, farmers, beekeepers, and the scientific community have reported an alarming decline of honey bees^2,3^. A wealth of empirical evidence attributes this phenomenon to a range of natural and anthropic stressors, including climate change, habitat degradation, intensification of farming, pesticides and increased pressure of pathogens and parasites^2,3^. In particular, the extensive use of agrochemicals in intensive crop farming is an increasing concern for scientists and beekeepers due to their detrimental impact on non-target insects, such as wild and managed pollinators and the fundamental ecosystem services they provide^2^. The debate about the danger posed by agrochemicals upon the environment, human health and beneficial species^4^, together with the ever-growing success of organic farming^5^, have renewed interest in undertaking more sustainable agricultural methods^6^.

Entomopathogenic fungi have been generally considered an effective and eco-friendly alternative to conventional chemical pesticides^7^. Among these, the fungus *Beauveria bassiana* (Balsamo) Vuillemin gained widespread popularity among users of commercial bioinsecticides (BPs), to the extent that it is currently found in more than a third of commercialized fungus-based products^8,9^. The success of *B. bassiana* can be mostly attributed to its ability to target a broad spectrum of pest species^10^. As for other entomopathogenic fungi, host infection occurs percutaneously: after penetrating the cuticle, fungal cells invade internal tissues, overcome the immune system of the host, proliferate by formation of hyphal bodies and finally kill the host^9,10^. In the final stage, the fungus outgrows from the dead host and produces new conidia that spread across the environment and infect other individuals^9,10^. *B. bassiana* is considered safe for humans and other non-target organisms as only negligible effects associated with its use have been reported so far^9^. In honey bees, mortality rate due to fungus exposure is considered negligible^9^. In addition, the majority of the existing field studies agreed upon the fact that *B. bassiana* does neither cause epizootic in managed hives nor has significant negative effects on whole colonies^9,11–14^. The lack of obvious toxicity for honey bees led some authors to propose the use of bee foragers as vectors to spread fungal spores across cultivations^14,15^ or the use of *B. bassiana* as miticide to control *Varroa* mite populations in managed hives^13,16^. Nevertheless, the alleged harmlessness of this fungus for pollinators and particularly for honey bees might be overestimated. Indeed, *B. bassiana* has been showed to sporulate on inoculated broods, causing a moderate, yet significant, increase in mortality^11,12^. Similarly, when inoculated in high doses, the fungus decreases longevity of adult workers^17^. In addition, a recent study reported that *B. bassiana* altered the cuticular profile of individual honey bees, disrupting nestmate recognition^18^. Altogether, these findings open up the possibility that this fungus may have non-negligible sublethal effects on honey bees, exacerbating the negative effects of other stressors and/or directly impacting the fitness of honey bee colonies^19,20^.

Assessing the side-effects of *B. bassiana* on honey bees is an urgent issue. Honey bees are particularly vulnerable to stressors and associated side effects due to their central-place foraging lifestyle^20^. Foraging requires a wide range of cognitive abilities^21,22^. Bees have to navigate a complex environment and be able to locate high-quality but variable and dispersed food sources. They must learn and remember the complex physical features and fragrances of the most rewarding flowers in order to communicate this information to nestmates once returned to the nest^21,23^. Foraging efficiency is achieved by behavioural plasticity^24,25^ and a subtle division of labour within the colony, which is finely regulated by temporal polyethism and pheromonal communication^26–28^. For instance, in honey bees there are at least three sub-groups of foragers: those collecting nectar, those collecting pollen and those specialized in collecting water^25,29^. One of the main determinants of such sub-division of labour is the individual sensitivity to sucrose^29^. Individual sucrose responsiveness determines the forager’s decision to exploit or ignore a certain food source and to recruit nestmates, thus affecting collective foraging decisions and foraging optimization^25^. Moreover, the subjective value of reward, reflected by the tendency to respond to sucrose solution, affects appetitive learning: indeed, highly responsive bees usually show better learning performance and stronger memory than low-responsive bees when sucrose is used as reinforcement^30–33^. As a result, even low-level behavioural impairments may undermine the ability of individual bees to fulfil their foraging task, and, ultimately, threat colony survival.

Considering that foraging depends to a large extent on sucrose responsiveness and appetitive learning and memory, we propose that evaluating the impact of the fungus on these capacities is an effective way to assess the sublethal effects of this biopesticide. In the present study, we made use of behavioural assays based on the proboscis extension response (PER), which is the reflexive appetitive response of tethered bees to an antennal sucrose stimulation^34,35^, to investigate whether *B. bassiana* impairs cognitive abilities of forager bees. In our first experiment, we evaluated whether fungus exposure affected longevity and food consumption in caged adult workers. Successively, we studied whether the fungus affected sucrose responsiveness, which may be assessed via the reflexive responsiveness of harnessed bees to sucrose solutions of increasing concentration. Then, we assessed whether *B. bassiana* affected habituation to antennal sucrose stimulation, which is usually negatively correlated with responsiveness to sucrose. Habituation is a case of non-associative learning^32^ underlying more complex forms of learning^36,37^, in which a non-reinforced response to a stimulus decreases after repeated presentations of that stimulus^32^. Finally, we used a PER conditioning procedure to study whether the fungus exposure affected appetitive associative olfactory learning and memory recall. PER conditioning procedures are extensively used in psychological, neurobiological and toxicological studies, and represent a standardized methodology to compare the impact of stressors on bees’ cognition^34^. Specifically, learning and memory functions can be evaluated using a classical (Pavlovian) conditioning procedure: a neutral stimulus (for instance an odorant) that produces no response in a bee is repeatedly paired with a reinforcement (i.e. sucrose, US) that reflexively elicits PER so that, at the end of the training, the previous neutral stimulus will be conditioned (CS) and will evoke the response by itself^35,38^.

## Results

### Mortality assay and energetic demand

**Figure 1:**
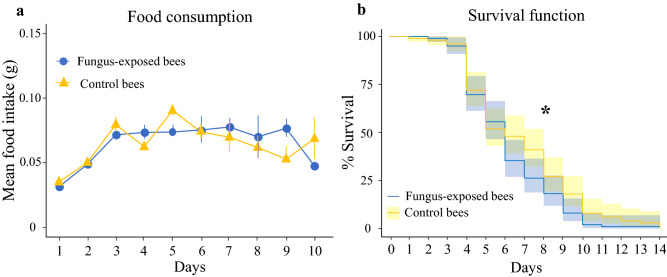
(**a**) *B. bassiana* does not affect sucrose consumption in caged foragers. Mean daily sucrose consumption of fungus-exposed (blue lines) and control bees (yellow lines) caged in groups of ten over 10 days. We analysed and displayed the food consumption only over 10 days as in the last 4 days the group of fungus-exposed bees counted only one individual. Data were normalized in function of the number of alive bees in each given day. No difference on sucrose consumption has been observed between groups (LMM, *treatment*: *p* = 0.72). (**b**) *B. bassiana* exposure affects survival longevity of foragers. Cumulative survival of fungus-exposed (n = 100) and control (n = 100) bees kept under caged conditions over 14 days. Fungus-exposed bees had significantly lower survivorship than controls (Log-rank Mantel Cox test, *p = 0.034). In particular, at the end of the 5th and the 6th day, a higher proportion of fungus-exposed bees died (χ^2^ test, 5th day: *p* = 0.045; 6th day: *p* = 0.017).

Honey bees facing infections might exhibit changes (increase/decrease) in their sucrose intake or diet to meet their nutritional and energetic demands^39,40^. We therefore assessed whether fungus-exposed (n = 100) and control bees (n = 100) differed in daily consumption of 50% sucrose solution during the 14 days of observation. In addition, as an increased mortality was previously observed in fungus-treated bees^17^, we investigated whether also our *B. bassiana* strain affected longevity of caged workers. The results showed that while sucrose consumption slightly increased over the course of the experiment (LMM, χ^2^ = 13.7, df = 1, *p* = 0.0002, Fig. 1a), fungus-exposed and control bees consumed an equal daily amount of food (LMM, χ^2^ = 0.12, df = 1, *p* = 0.72, Fig. 1a, SM1). Survival censuses showed that the median lethal time (LT50) did not differ among groups, being in both cases between the 4th and the 5th day from exposure (LT50 at the 4th day: 54% fungus-exposed bees vs. 53% control bees; LT50 at the 5th day: 34% fungus-exposed bees vs. 49% control bees). However, at the end of the 5th day, a higher proportion of fungus-exposed bees died (χ^2^ test, χ^2^ = 4.022, *p* = 0.045, Fig. 1b). The difference in the mortality rate among groups was even more evident at the end of the 6th day (χ^2^ test, χ^2^ = 5.70, *p* = 0.017, Fig. 1b). A finer statistical evaluation of the survivorship of fungus-exposed and control bees revealed that the fungus was a significant predictor of mortality (Log-rank Mantel Cox test, Z =  − 2.11, df = 1, *p* = 0.034, Fig. 1b). Furthermore, the factor “cage” had no effect on bee mortality (Log-rank Mantel Cox test, Z = 1.31, df = 1, *p* = 0.19). According to these results all behavioural experiments were performed 3 days after fungus exposure, a time in which the fungus was sublethal for bees^9,18^.

### Sucrose responsiveness

**Figure 2:**
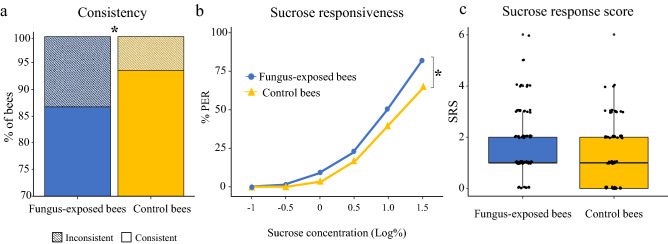
(**a**) *B. bassiana* exposure affects consistency of appetitive responses in honey bees. Percentage of fungus-exposed (n = 21) and control (n = 10) bees that exhibited inconsistency (i.e. showed PER to lower, but not to higher, concentrations of sucrose) when presented with the series of six sucrose solutions of increasing concentration (0.1%, 0.3%, 1%, 3%, 10% and 30% w/w). The fungus-exposed group had a significantly higher proportion of inconsistent bees (twice as many controls) than the control group (χ^2^ test, *p = 0.035). (**b**) *B. bassiana* exposure affects sucrose responsiveness. Cumulative proportions of fungus-exposed (blue) and control (yellow) bees displaying PER when presented with six sucrose solutions of increasing concentration. Bees treated with *B. bassiana* (n = 86) were significantly more responsive to sucrose than untreated bees (n = 92), (GLMM, *treatment*: *p* = 0.011). (**c**) *B. bassiana* did not affect sucrose response scores. Median, quartiles, minimum and maximum sucrose response scores (SRS) values of fungus-exposed (n = 86) and control (n = 92) bees. Dots represent SRS of individual bees. Individual SRS was calculated as the number of PER to sucrose solutions exhibited by the bee over the test. No difference has been found in the overall SRS between the two groups (Mann–Whitney *U* test, *p = 0.065).

Individual sucrose responsiveness is tightly linked to the fine division of labour within the forager sub-caste^25,29^ and it represents one of the main predictors of cognitive performances when sucrose is used as reinforcement^30–33^. Thus, we investigated whether fungus exposure affected the sucrose responsiveness of harnessed forager bees using a standard procedure^41,42^. Fungus-treated (n = 120) and control bees (n = 114) were sequentially stimulated on the antennae with six sucrose solutions of increasing concentration (0.1%, 0.3%, 1%, 3%, 10% and 30%), each interspersed with a water stimulation to avoid antennal sensitization. Bees that never responded during the test were presented with a final 50% sucrose solution to induce PER, and non-responders discarded. An individual sucrose response score (SRS) was calculated as the number of PER to sucrose solutions exhibited by each bee over the test. A first comparison between groups revealed that the fungus caused a significant increase in the number of bees showing inconsistent responses (i.e. bees that responded to a lower but not to a higher sucrose concentration (fungus-exposed bees (17.5%), control bees (8.7%); χ^2^ test, χ = 4.3, *p* = 0.035, Fig. 2a). For a more rigorous approach, we excluded from the analysis all inconsistent subjects and all those that responded to water stimulations (fungus-exposed bees, 17.5%; control bees, 15.8%)^42^. A preliminary analysis showed no difference between groups in the proportion of bees responding to water (χ^2^ test, χ = 0.3, *p* = 0.56). As expected, the proportion of PER to sucrose solutions significantly increased with increasing sucrose concentrations in both groups (GLMM, Z = 9.01, *p* < 0.0001, Fig. 2b). Yet, fungus-exposed bees (n = 86) exhibited a significantly higher sucrose responsiveness than controls (n = 92) (GLMM, Z =  − 2.55, *p* = 0.011, Fig. 2b, SM2). Accordingly, a significantly lower proportion of fungus-exposed bees did not respond to any sucrose solution but to the final 50% (χ^2^ test, χ = 4.85, *p* = 0.027). A similar trend, although not statistically significant, was found in the SRS, which provides an individual assessment of sucrose responsiveness (Mann–Whitney *U* test, W = 4569, *p* = 0.065, Fig. 2c).

### Habituation

**Figure 3:**
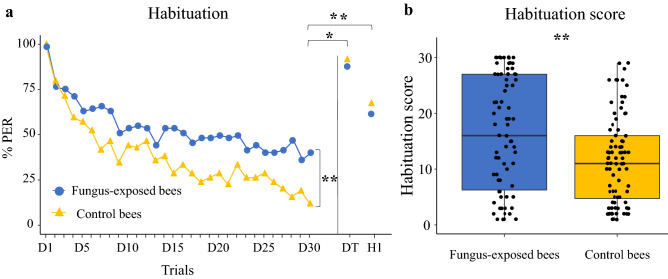
(**a**) *B. bassiana* exposure affects habituation of appetitive responses. Habituation curves of fungus-exposed (n = 74) and control bees (n = 84). Habituation was assessed by stimulating bees on the antennae 30 consecutive times with 10% sucrose solution. After 10 s from the last habituation trial, bees were stimulated with 50% sucrose solution to induce dishabituation (DT). After an additional 10 s, bees were stimulated again with the original habituation stimulus to check for response recovery following dishabituation (H1). Bees treated with the fungus showed a higher resistance to extinction than control bees (GLMM, *treatment*: **p = 0.0007). Both groups recovered when presented with the DT and responded to the original H1, thus confirming a true habituation phenomenon. No difference in responses to the DT (Wilcoxon test, *p* = 0.27) and to the H1 (Wilcoxon test, *p* = 0.12) was observed between the two groups. (**b**) *B. bassiana* exposure affected Habituation Scores. Median, quartiles, minimum and maximum Habituation Score (HS) values. Dots represent individual bees’ HS values. Individual Habituation scores (HS) were calculated as the number of PER exhibited by the bee over the 30 habituation trials. Fungus-exposed bees had a significantly higher HS than controls (Mann–Whitney *U* test, **p = 0.0035).

Our next investigation focused on whether bees exposed to the fungus exhibited alterations in antennal sucrose habituation. To this aim, fungus-exposed (n = 74) and control bees (n = 84) were stimulated thirty times on their antennae with 10% sucrose solution. The number of sucrose stimulations eliciting PER in each bee was used to compute an individual habituation score (HS), which represented the individual degree of habituation and ranged from 1 to 30. Habituation is usually negatively correlated with sucrose responsiveness: bees that are highly responsive to sucrose usually exhibit a higher resistance to extinction of appetitive responses than low responsive bees^32^. Thus, since fungus-exposed bees had a higher sucrose responsiveness in our previous assay, we expected them to show a lower degree of habituation than controls. Both groups of bees showed a significant decrease in PER frequency over 30 consecutive sucrose antennal stimulations, thus exhibiting habituation (GLMM, *trial*: χ^2^ = 544.88, df = 1, *p* < 0.0001, Fig. 3a). The group of fungus-exposed bees exhibited a higher resistance to extinction of PER than the control group (GLMM, *treatment*: χ^2^ = 11.52, df = 1, *p* = 0.0007, Fig. 3a, SM3). Furthermore, individual fungus-exposed bees had a significantly higher HS than controls (Mann–Whitney *U* test, W = 3946, *p* = 0.0035, Fig. 3b). To verify whether this result was due to true habituation and not to sensory adaptation and/or motor fatigue, 10 s after the last habituation trial bees received a single antennal 50% sucrose stimulation to induce dishabituation (Dishabituation trial, DT). To check whether dishabituation has occurred, after 10 additional seconds, bees were stimulated again with the original stimulus (i.e. 10% sucrose solution, H1). In both groups PER to the dishabituation stimulus was significantly higher than the responses observed in the last habituation trial (Wilcoxon test, fungus-exposed bees: Z =  − 6.00, *p* < 0.0001, control bees: Z =  − 8.31, *p* < 0.0001, Fig. 3a). The increased response to the novel stimulus (i.e. 50% sucrose solution) is consistent with the notion of stimulus specificity of habituation^36^. Accordingly, the sucrose responses to the original habituating stimulus after the DT were significantly higher than those observed in the last habituation trial (Wilcoxon test, fungus-exposed bees: Z =  − 2.86, *p* = 0.004; control bees: Z =  − 6.93, *p* < 0.0001, Fig. 3a). We did not observe any difference between groups in responses to the dishabituation stimulus (Mann–Whitney *U* test, W = 2957, *p* = 0.27) nor to the original habituating stimulus after DT (Mann–Whitney *U* test, W = 2736, *p* = 0.12, Fig. 3a). These data ruled out sensory adaptation, motor fatigue or other alternative explanations for the observed decrease in PER over the 30 trials, thus confirming a true habituation phenomenon.

### Associative learning

**Figure 4:**
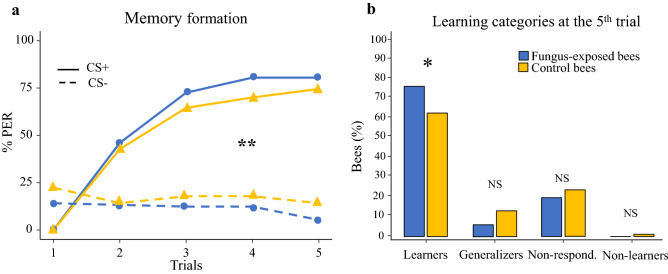
(**a**) *B. bassiana* exposure modulates associative olfactory learning. Proportions of PER to rewarded (solid lines) and unrewarded (dotted lines) odorants exhibited by fungus-exposed (n = 113) and control (n = 110) bees over the five conditioning trials. Fungus-exposed bees performed better than controls in the associative learning task (GLMM, treatment*CS: **p < 0.0001). In particular, fungus-exposed bees responded less to the unrewarded odorant (*CS*−) than control bees, whereas no difference was observed in PER to the rewarded stimulus (*CS* +) (GLMM, Dunnett’s post hoc test, *CS*−: *p* = 0.081; *CS*+: *p* = 0.54). (**b**) *B. bassiana* exposure enhanced specific learning at the 5th conditioning trial in bees. Percentage of fungus-exposed and control bees belonging to each of the four possible learning categories on the 5th conditioning trial. A significantly higher proportion of fungus-exposed bees was found in the specific learners category *(*χ^2^ test, *p = 0.03), whereas no difference was observed in the proportion of fungus-exposed and control bees in the other categories (χ^2^ test, *Generalizers*: *p* = 0.053: *Non-responding*: *p* = 0.45: *Non-learners*: *p* = 0.2).

A higher sucrose responsiveness is usually positively correlated with learning and memory performances when sucrose is used as reinforcement^30–33^. Thus, given the interference of the pathogen with sucrose responsiveness and habituation, we also expected an impact on learning and memory retention performance. To test this prediction, 3 days after treatment, bees were subjected to a differential conditioning procedure in which they had to learn to discriminate between two olfactory stimuli. In the training phase, bees experienced five pairings of an odorant (CS +) with 30% sucrose solution (US) and five presentations of an alternative odorant (CS−) without reward. For each bee, an individual acquisition score (ACQS) was calculated as the number of PER exhibited over the five reinforced trials. Note that the individual ACQS could range from 0 (i.e. bees that never responded to the CS +) to 4, as the bees responding to the first CS+ presentation were discarded from the training. Both fungus-exposed bees (n = 113) and controls (n = 110) successfully learned to discriminate between the stimuli over the course of the conditioning phase (GLMM, *trial*: χ^2^ = 112.99, df = 1, *p* < 0.0001, Fig. 4a, SM4). Responses to the CS+ and the CS− were significantly different at the end of the training in both groups (GLMM, *CS*: χ^2^ = 231.83, df = 1, *p* < 0.0001, Fig. 4a). Moreover, a significant interaction was found between the treatment and the CS (GLMM, treatment*CS interaction: χ^2^ = 15.78, df = 1, *p* < 0.0001, Fig. 4a). While the two groups of bees did not differ in their response to the CS+ and the CS− (GLMM, Dunnett’s post hoc test, *CS*− : Z =  − 2.39, *p* = 0.081; *CS*+: Z = 1.37, *p* = 0.54, Fig. 4a), overall, a higher proportion of fungus-exposed bees were specific learners (i.e. responded to the CS+ and not to the CS−)^38^ at the 5th trial (χ^2^ test, χ = 4.65, *p* = 0.03, Fig. 4b) than controls. Nonetheless, the impact of the fungus on memory formation was quite weak as, at the individual level, fungus-exposed bees did not show a higher ACQS than controls (Wilcoxon test, W = 6851, *p* = 0.17, Fig. S1). No difference was found in any of the other possible categories of unsuccessful learners belonging to the two groups of bees (χ^2^ test, *Generalizers*: responding to both the CS+ and the CS− , χ = 3.76, *p* = 0.053; *Non-responding:* χ = 0.57, *p* = 0.45; *Non-learners:* responding to the CS− but not to the CS+, χ = 1.96, *p* = 0.2, Fig. 4b).

**Figure 5:**
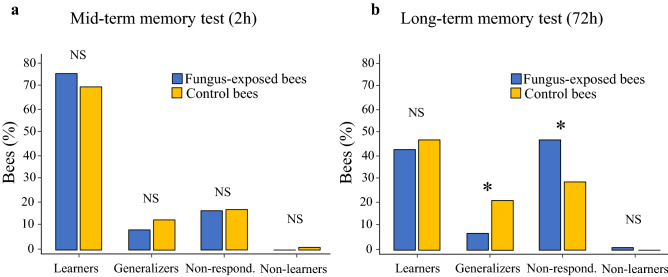
(**a**) *B. bassiana* exposure did not affect short-term memory performances. Percentage of fungus-exposed (n = 110) and control (n = 105) bees showing specific memory (i.e. responded to the CS+ and not to the CS−) and not (all other possible combinations) in the test performed 2 h after conditioning. No significant difference was observed in the proportion of fungus-exposed and control bees that showed specific memory (χ^2^ test, *p* = 0.35). Similarly, the proportion of all other categories of unsuccessful learners differed between fungus-exposed and control bees (χ^2^ test, *Generalizers*: *p* = 0.32; *Non-responding: p* = 0.9; *Non-learners: p* = 0.31). (**b**) *B. bassiana* exposure does not affect long-term memory performances in bees treated after conditioning. Bees were first trained in an appetitive olfactory learning. Bees that learned at the 5th conditioning trial (i.e. responded to the CS+ and not to the CS−) were either exposed to *B. bassiana* (n = 79) or treated with sterile water (n = 67) and successively tested for memory retrieval after 72 h. Specific long-term memory did not differ between fungus-exposed and control bees (χ^2^ test, *p* = 0.57). However, among unsuccessful learners, a higher proportion of fungus-treated bees were *Non-responding* (χ^2^ test, *p* = 0.025), while a higher proportion of control bees were *Generalizers* (χ^2^ test, *p* = 0.011)*. Non-learners* were equally represented in both group of bees (χ^2^ test, χ = 0.85, *p* = 0.36). (*p < 0.05).

We finally evaluated whether *B. bassiana* exposure modulated mid-memory retention (2 h) by presenting the two odours in a random order without any reward. Again, to evaluate memory retention, we quantified the proportion of learners (i.e. bees with specific memory) and of the three different categories of unsuccessful learners (i.e. *Generalizers*, *Non-learners* and *Non-responding bees*). Despite fungus-exposed bees (n = 110) performing better in the acquisition phase than controls (n = 105), there was no significant difference between groups in specific memory performance (χ^2^ test, χ = 0.88, *p* = 0.35, Fig. 5a) nor in performance of the unsuccessful learners (χ^2^ test, *Generalizers*: χ = 0.99, *p* = 0.32; *Non-responding:* χ = 0.02, *p* = 0.9; *Non-learners:* χ = 1.04, *p* = 0.31, Fig. 5a).

### Long-term memory retrieval

In the previous experiment, bees that underwent the memory test were exposed to the fungus at the time of the training. To test whether fungus exposure specifically affected long-term memory retrieval, we used the same olfactory conditioning procedure described above to train untreated bees. Bees (n = 146) that successfully responded to the CS+ and not to the CS− in the last conditioning trial were randomly assigned to either fungus-exposed or control group and treated accordingly. After 72 h, fungus-exposed (n = 79) and control (n = 67) bees were presented again with the CS+ and CS− in random order without reward. When we quantified for each group the proportion of bees that showed specific memory no difference was found in memory retrieval performance at 3 days (χ^2^ test, χ = 0.33, *p* = 0.57, Fig. 5b). However, among unsuccessful learners, a higher proportion of fungus-treated bees were *Non-responding* (χ^2^ test, χ = 5.04, *p* = 0.025, Fig. 5b), while a higher proportion of control bees were *Generalizers* (χ^2^ test, χ = 6.44, *p* = 0.011, Fig. 5b)*. Non-learners* were equally represented in both group of bees (χ^2^ test, χ = 0.85, *p* = 0.36, Fig. 5b). Therefore, fungus-exposed bees were generally less responsive to odorants than control bees.

## Discussion

Recent evidence shows that sublethal exposure to chemical pesticides and pathogens induces a variety of side effects on wild and managed pollinators, threatening their fitness and survival^20,43–45^. In the present study, we investigated whether the entomopathogenic fungus *B. bassiana*, which is widely used as biopesticide, interfered with cognition in honey bee foragers. To this end, we used the well-established PER protocol to assess whether *B. bassiana* affected individual responsiveness to sucrose, habituation of appetitive responses, associative olfactory learning and mid-term and long-term memory. An initial survival assessment demonstrated that *B. bassiana* reduced caged workers longevity over 14 days of observation, confirming previous reports^17^. In particular, a significant increase in mortality in the fungus-exposed group was observed after 5–6 days from treatment. Since our behavioural assays were performed on the 3rd day after treatment, we exclude that our findings can be explained by a reduction in vitality of bees. Here, we demonstrate that fungus-treated bees displayed an increased responsiveness to sucrose. Moreover, fungus-exposed bees exhibited a significantly higher resistance to extinction of appetitive responses to sucrose and, surprisingly, a better performance in the olfactory learning phase than controls. Yet, *B. bassiana* exposure did neither positively nor negatively affect mid- and long-term memory.

Fungus-exposed and control bees did not differ in sucrose consumption during almost 2 weeks of observation. This suggests that the enhanced responsiveness of fungus-exposed bees could not be explained by increased energetic demand or metabolic stress, as previously reported for other pathogens^39,40^. Since *B. bassiana* exposure alters honey bee cuticular chemical profile^18^, an alternative hypothesis would be that the enhanced responsiveness is caused by desiccation. However, all bees were provided with water ad libitum before the test and all water-responding bees were discarded from the analysis. In addition, we found no difference in the proportion of bees responding to water between the two groups, thus ruling out the possibility that dehydration played a major role in our assay.

Interestingly, sucrose responsiveness did not change linearly with the logarithmic increase of sucrose concentration. The difference between the two groups was most pronounced in the responses to the highest concentrations of sucrose suggesting that the fungus over motivated bees for sucrose. Thus, in the field, these bees might dance more vigorously after returning to the hive. Recruitment dances become more frequent and rapid with increasing profitability of a food source, for instance, in its sugar concentration^46–48^. As a result of the over motivation of dancers, the expectations generated in the recruited bees may not be met by the rewards found in the field, causing recruited bees to return to the nest without collecting food.

Strikingly, fungus-exposed bees were also twice as inconsistent than controls in the sucrose responsiveness test, displaying PER to sucrose solutions at lower but not at higher concentrations. While it remains unclear whether this was due to motor impairments, deficits in sucrose perception or other unidentified processes, such impairments may undermine the ability of foragers to assess the actual reward provided by flowers, thus affecting their foraging efficiency. Overall, these findings may raise concerns, since individual sucrose responsiveness is closely related to the division of foraging tasks within the colony^25,29^. Highly responsive foragers are more likely to collect pollen or water, whereas the least responsive ones selectively go for nectar^25,29^. As a result, the quality and the quantity of pollen and nectar available to the colony at any given time depend, among other factors, on the assortment of sucrose response thresholds within the cohort of foragers. *B. bassiana* exposure might unsettle the numerical ratio between the sub-castes of foragers and, consequently, affects the balance of different nutrients available to the colony.

Our experiments also demonstrate that fungus-exposed bees displayed a higher resistance to extinction of appetitive responses than controls. This finding is not surprising given that sucrose responsiveness is closely linked to non-associative learning performances^30,49^. Highly responsive bees usually exhibit a lower degree of habituation of PER than low responsive ones, and *vice-versa*^50^. This phenomenon can be explained by the fact that bees differing in sucrose responsiveness also differ in their subjective evaluation of the reward^30^. Therefore, the higher resistance to extinction of PER in fungus-exposed bees can be explained by their increased sucrose responsiveness and more positive subjective evaluation of the perceived reward.

Fungus-treated bees also exhibited a better acquisition performance in the appetitive olfactory learning task than controls. This finding is consistent with the results obtained in our habituation and responsiveness assays. The relationship between sucrose responsiveness and associative learning has been widely demonstrated^31,51,52^, and the role of US strength in learning is well-known^53,54^. In honey bees, these functions depend to a great extent on molecular processes mediated by biogenic amines, in particular by octopamine^55^. Our behavioural observations suggest that fungus exposure may either directly or indirectly interfere with the octopaminergic signalling cascade within the bee nervous system. A pharmacological approach aimed at specifically blocking/activating octopaminergic receptors might help to shed light on the molecular underpinnings of *B. bassiana* action.

Conversely, fungus exposure did not affect mid-term memory recall performances. This result contrasts with the established notion that bees exhibiting higher learning scores also perform better in memory retention tests^30,33^. Other pathogens have also been found to uncouple the tightly linked physiological processes underlying habituation, sucrose responsiveness and appetitive learning and memory in bees^44^. For instance, honey bees infected by the deformed wing virus showed an increased responsiveness to sucrose, an impaired associative learning and memory formation, but intact sensitization and habituation^44^. While the physiological mechanisms targeted by pathogens have not been yet identified, the activation of the immune system is likely to play a central role. Indeed, even a non-pathogenic activation of the immune system leads to impairments in associative learning and memory in bees^56,57^. However, a more conservative account for our results is that difference between fungus-exposed and control bees in learning was too modest to induce differences in the memory test. Alternatively, it might be that retrieval per se is unaffected and that it is the establishment of the associative link that is promoted by the fungus. This latter hypothesis is corroborated by our long-term memory assay, in which all bees had an identical learning level prior to the test (see Methods). Again, we did not find any significant difference in long-term specific memory between the two groups, demonstrating that *B. bassiana* did not specifically affect memory retrieval or its underlying neural mechanisms. Interestingly, a closer look to the results showed that fungus-exposed bees were under-represented in the “*generalizers*” category and over-represented in the “*non-responders*” category. This suggests that fungus-exposed bees were generally less likely to respond to odorants or less motivated to attempt a random response during the test. This might also result from deficits in the odour coding occurring at the level of the antennal lobes. We suggest that in vivo optical imaging applied to these brain regions might be a further step to shed light on the mechanisms underlying *B. bassiana* action.

Taken together, our findings suggest that *B. bassiana* might have a stronger impact on honey bees than previously thought. Previous studies often overlooked potential side effects of the fungus, especially on individuals’ behaviour and cognition. A recent study^18^ reported that fungus-treated bees were more likely to be accepted in alien colonies, potentially favouring drifting phenomena and, in turn, the spread of pathogens and parasites. Treatments with *B. bassiana* reduced birth weight and increased mortality in brood^16^ and our study confirmed the pathogenicity of *B. bassiana* in adult bees^17,58^. Further studies should aim at clarifying whether *B. bassiana* treatments affect foraging efficiency of bees in realistic field conditions and further effort should be made to elucidate the mechanisms of action of this biopesticide. More importantly, to date it is unknown whether fungal treatments may reduce the ability of bees to withstand other simultaneous stressors such as pathogens or pesticides. An additional key open question to address remains whether fungus-exposed bees also have compromised ability to taste key nectar constituents such as secondary metabolites, which also modulate pollinators’ cognition and behaviour^59–62^. A must for a critical and efficient risk assessment of biopesticides should also consider their impact on wild pollinators, too often neglected. Although the use of natural pathogens and other biocontrol strategies^7^ is certainly to be pursued and preferred over chemical pesticides, a careful assessment of their potential side-effects on wild and managed beneficial species is essential before claiming their complete safety. Aside from classical toxicological assays, particular attention should be paid to the investigation of the impact of biocontrol agents on behavioural traits that are crucial to maintain the complex social organization of honey bees^63,64^.

## Methods

### *Beauveria bassiana*

Infective conidia of *Beauveria bassiana* (Balsamo) Vuillemin were isolated and collected by plating 100 µl of the commercial bioinsecticide *Naturalis* (Intrachem Bio International S.A.) on Petri dishes with MEA (Malt Extract Agar). *Naturalis* is a concentrated suspension of at least 2.3 × 10^7^ spores/ml of the naturally occurring *B. bassiana* strain ATCC 74.040. After 3 days of incubation at 30 °C, emerging spores were collected and re-suspended in a 0.01% Triton-X-100-water solution at a concentration of 10^9^ spores/ml^18^. Spore concentration was assessed through vital count after plating 100 µl of serial dilutions of the fungal solution on Petri dishes with Yeast Extract–Peptone–Dextrose (YPD) incubated for 3 days at 30 °C. The fungal solution at 10^9^ spores/ml was used to expose bees via topical application throughout all experiments. In all assays, the experiment was performed 3 days after treatments, a period sufficient for *B. bassiana* to germinate, penetrate the cuticle and develop inside the body of the host^9^.

### Mortality assay

We initially investigated the impact of *B. bassiana* exposure on honey bees’ survival and sucrose consumption. As described in the standard methods for toxicology research in *A. mellifera*^65^, after being collected and treated with either the fungus or the solvent solution (see general methods), bees were housed in 120 ml Plexiglas cages in groups of 10 individuals each. Each container was equipped with a 20 ml syringe providing 50% sucrose solution ad libitum. Syringes were deprived of the cone luer to provide the bees an easy access to the food and, at the same time, to prevent any sugar solution leakage. Ten cages housing 10 bees each were used for each group, for a total of 20 cages. To control for solution evaporation, an additional syringe was mounted in an empty cage and maintained under identical conditions over the entire course of the experiment. Dead bees were counted and syringes were weighted daily to quantify mortality and sucrose consumption respectively. Daily sucrose consumption was normalized in function of the number of alive bees in each container at the given day.

### Behavioural assays

#### General methods

Honey bee foragers (*Apis mellifera ligustica*) were collected while leaving a feeder providing 50% sucrose solution (w/w) placed approximatively 10 m apart from an apiary consisting of six colonies (Department of Biology, University of Florence). Bees were immediately brought to the laboratory, cold anaesthetized for approximatively 5 min and randomly assigned to one of the experimental groups. Bees were treated via topical application on the scutum (thorax) with either 1 µl of the *B. bassiana* solution (10^6^ spores) (fungus-exposed bees) or 1 µl of the solvent alone (sterile water + Triton) (controls)^18^. After treatment bees were housed in groups of 50 individuals in small plexiglass cages (9 × 7 × 11 cm) and provided with 50% sucrose solution ad libitum. Cages were maintained at room temperature (24 ± 2 °C) and 50 ± 10% humidity. Two days later, surviving bees were removed from cages, cold anesthetized and individually harnessed in plastic tubes by placing a small strip of duct tape in between the head and the thorax. Their heads were fastened to the holder with a drop of low-temperature melting wax so that the bees could freely move only their antennae and mouthparts^38^. After recovery (approx. 30 min), all bees were fed ad libitum with 50% sucrose solution to equalize the level of hunger and kept resting overnight in a dark and humid place at room temperature (24 ± 2 °C). Proboscis extension response (PER) can be elicited by stimulating restrained bees on the antennae with sucrose solution. The following day bees were tested according to the experimental condition (see below). In the long-term memory assay (see below), bees were first harnessed and conditioned and then infected. All experiments were carried out between April and July 2019 under controlled laboratory conditions at the Department of Biology of the University of Florence (Italy).

### Sucrose responsiveness

Before the sucrose responsiveness assay, we controlled as much as possible for the effect of thirst on sucrose responsiveness^42,66^. To this end, all bees were repeatedly stimulated with water and were allowed to drink it ad libitum when necessary to reduce at minimum the possibility that bees would have showed PER to water and not to the sucrose dissolved in it. At the beginning of the test, an additional stimulation with water was provided^42^ and responding bees were immediately discarded. Afterward, we quantified the sucrose responsiveness of restrained bees by presenting them with six sucrose solutions of increasing concentration: 0.1%, 0.3%, 1%, 3%, 10% and 30%, with an inter-stimulus interval (ISI) of 2 min. Each sucrose presentation was interspersed with a water stimulation to avoid sucrose sensitization. Bees that never responded during the test were presented with a final 50% sucrose solution to induce PER. Bees that never showed PER to a final 50% sucrose solution stimulation were discarded from the analysis because the lack of response to such a higher sucrose concentration may be due to a motor problem. To control for the effect of thirst on the sucrose responsiveness, bees that responded to water stimulations were discarded from the analysis^42^. Finally, bees that showed inconsistency in the responses (i.e. responded to a lower but not to a higher concentration of sucrose) were also discarded from the analysis, since the lack of response may be due to other uncontrolled factors than sucrose threshold^42^. PER occurrence was recorded for each trial and an individual Sucrose Responsiveness Score (SRS) was computed as the number of PER showed over the six sucrose stimulations. Therefore, SRS could range from 0 to 6.

### Non-associative learning assay (habituation)

In the training phase, harnessed bees (74 fungus-exposed bees and 84 controls) received 30 consecutive short antennal stimulations (less than a second) with 10% sucrose solution and with an inter-trial interval (ITI) of 10 s^32^. Occurrence of PER (Yes/No) was recorded at each stimulation and a final individual Habituation Score (HS) was calculated as the number of PER showed over the 30 stimulations by each bee. Bees that did not respond to the first stimulation was discarded, so the HS could range from 1 to 30. After 10 s from the last trial, we induced dishabituation in the bees by providing one additional antennal stimulation with 50% sucrose solution (dishabituation trial, DT). To observe whether dishabituation has occurred, bees were presented again with the original stimulus used in the training phase (10% sucrose solution) 10 s after the dishabituation trial. This allowed us to rule out alternative explanations for the decrease in PER to antennal stimulations, such as sensory adaptation or fatigue.

### Olfactory learning and mid-term memory retention

The proboscis extension response (PER) can be conditioned through a classical Pavlovian conditioning procedure^35,67^. In this protocol, a conditioned stimulus (CS, i.e. an odour) is presented repeatedly in close temporal association with an unconditioned stimulus (US, i.e. sucrose delivered to the antennae) that reflexively elicits the PER and a reward (sucrose solution delivered to the proboscis). When the association is established the conditioned stimulus will elicit PER by itself^35^. In our experiment we used an appetitive differential conditioning procedure: bees experienced five pairings of an odorant (CS +) with 30% sucrose solution to antennae (US) and mouthparts (reward) and five presentations of an alternative odorant (CS−) without sucrose. Thus, subjects had to learn to respond to the CS+ and to not respond to the CS− ^35,38^. We chose 1-hexanol and nonanal as odorants, since there is low response generalization between them^68^. Bees that showed PER to the first presentation of the CS+ were immediately discarded as learning could not be addressed. We balanced the experimental design so that the reinforced odorant (CS +) was 1-hexanol for half of the bees in each group and nonanal for the other half. Odorant presentations were spaced by 10 min and were pseudorandomized according to their contingency (i.e. rewarded vs. unrewarded)^38^. The odorants were provided to the bees by an odour releaser controlled by the microcontroller board Arduino Uno. Subjects were sequentially tested in groups of 20 each. At the beginning of the training, a bee was placed 4 cm in front of the odour releaser providing a continuous airflow of clean air. After 10 s of familiarization, a CS (either 1-hexanol and nonanal) was provided for 4 s and continuously carried away through a hole behind the bee by an exhaust system. In the reinforced trials, after 3 s from the odorant onset the bee received an antennal stimulation with sucrose by means of a toothpick and was allowed to drink for 3 s. No punishment was provided in the unrewarded trials. After 10 s of resting the bee was removed from the apparatus and another subject processed. For each individual, PER occurrence during the CS-only presentation was recorded over the ten conditioning trials. After conditioning, bees were kept in a dark place and tested 2 h later for memory retention. Only a small percentage of bees died before memory retention test (less than 4%). During memory test, odorants were provided without any reward. Moreover, the order of the presentation of the CS+ and the CS− was randomized between bees. To evaluate memory retention, we quantified the proportion of bees in each group that correctly responded to the CS+ and not to the CS− , namely bees with specific memory^38^. Bees that never responded during the memory retention test received an additional antennal stimulation with 50% sucrose solution to verify for their motivation or physical conditions. Bees that did not showed PER to this last stimulation were discarded from the analysis.

### Long-term memory retrieval

To investigate whether *B. bassiana* specifically affects memory processes, we used an alternative procedure. Bees were caught while landing at the feeder to select individuals with an empty crop. After being caught they were immediately cold immobilized and individually harnessed in the plastic tubes. After recovery (approx. 30 min), bees were fed with 5 µl of 50% sucrose solution and placed in a dark place to let them habituate to the harnessed condition. Two hours later, we trained bees using the same appetitive differential conditioning procedure as described above. We recorded PER occurrence during the CS-only presentation over the 10 conditioning trials and only subjects that successfully learned the association (“learners”, i.e. bees that correctly responded to the CS+ and not to the CS− in the last conditioning trial) were selected whereas the others were discarded. After conditioning, half of the learners received 1 µl of 10^−6^ *B. bassiana* solution on their scutum (thorax), whereas the other half received 1 µl of the solvent solution (sterile water + Triton). Harnessed bees were then kept resting in a dark place and fed twice a day (morning and late afternoon) with 15 µl of 50% sucrose solution. Three days later, bees were tested for long-term memory retrieval. In this phase bees were presented with the odorants without providing any reward. The order of the presentation of the CS+ and the CS− was randomized between bees. PER occurrence was recorded during the test. Bees that did not show PER during the memory test received an additional antennal stimulation with 50% sucrose solution to verify for their motivation and/or physical conditions. Bees that did not showed PER even to this last stimulation were discarded from the analysis^38^.

### Statistical analysis

χ^2^ tests was used to compare frequencies of inconsistent bees or bees responding to water in fungus-exposed and control groups. In both the sucrose responsiveness assay and the non-associative learning assay, response to sucrose solution (PER: 1/0) of individual bees was examined using an ANOVA design. Precisely, we ran a series of generalized linear mixed models (GLMMs) with a binomial error structure—logit-link function, *glmer* function of R package *lme4*. When necessary, models where optimized with the iterative algorithm BOBYQA. In the model run for the sucrose responsiveness assay “*bee response*” was entered as dependent variable, ‘*treatment*’ was entered as fixed factor and “*sucrose concentration*” as a covariate. In the model run for the habituation assay “*bee response*” was entered as dependent variable, ‘*treatment*’ as fixed factor and “*stimulation trial*” as a covariate. Mann–Whitney *U*-test was used to further compare SRS and HS between fungus-exposed and control bees. Wilcoxon test was used to compare the last habituation trial (D30) to the Dishabituation trial (DT) and to the original habituation stimulus (H1) within each group of bees. Appetitive olfactory learning performance of fungus-exposed and control bees was analysed using (GLMMs) with a binomial error structure—logit-link function (see above)*.* In the models for acquisition ‘*bee response*’ was entered as dependent variable, ‘*treatment*’ and ‘*CS*’ as fixed factors, and ‘*conditioning trial*’ was used as covariate. In all models the individual identity “*IDs*” was entered as a random factor to account for repeated measures. Interactions were evaluated in all full models and reported when significant. In all models, we retained the significant model with the highest explanatory power (i.e., the lowest AIC value). In the mortality assay, sucrose consumption was evaluated with a linear mixed model (LMM) where “*sucrose consumption*” was entered as dependent variable, ‘*treatment*’ as fixed factor and “*day*” as a covariate. “*Cage identity*” was entered as a random factor to account for repeated measures. For the statistical evaluations in the survival experiments, we ran the Breslow Statistic (Mantel–Cox Test) using the *Cox* function of R package *Survival*. In the regression model were entered the variables “*cage identity*” and “*treatment*”. All analyses were performed with R 3.4.2.

## Supplementary information

Supplementary Information.

## Data Availability

Data are available upon reasonable request to the corresponding authors.
