# Supplementary material for: Exposure to a biopesticide interferes with sucrose responsiveness and learning in honey bees
Source: Sci Rep. 2020 Nov 16;10:19929. doi: 10.1038/s41598-020-76852-2 (PMC7670424; doi:10.1038/s41598-020-76852-2)
Supplement: Supplementary file 1 — Supplementary Information. [file 41598_2020_76852_MOESM1_ESM.docx]

**Supplemental Information**

**A biopesticide exposure interferes with sucrose responsiveness and learning in honey bees**

Daniele Carlesso^1, 2^, Stefania Smargiassi^3^, Lara Sassoli^1^, Federico Cappa^1^, Rita Cervo^1^, David Baracchi^1^*.

*^1^ Department of Biology, University of Florence, Via Madonna del Piano, 6, 50019 Sesto Fiorentino, Italy*

*^2^ Department of Biological Sciences, Macquarie University, Sydney, NSW 2109, Australia*

*^3^ Department of Life Sciences and Systems Biology, University of Turin, Via Accademia Albertina 13, Turin 10123, Italy*

***Correspondence to** David Baracchi ([*david.baracchi@unifi.it*](mailto:david.baracchi@unifi.it)*)*

***
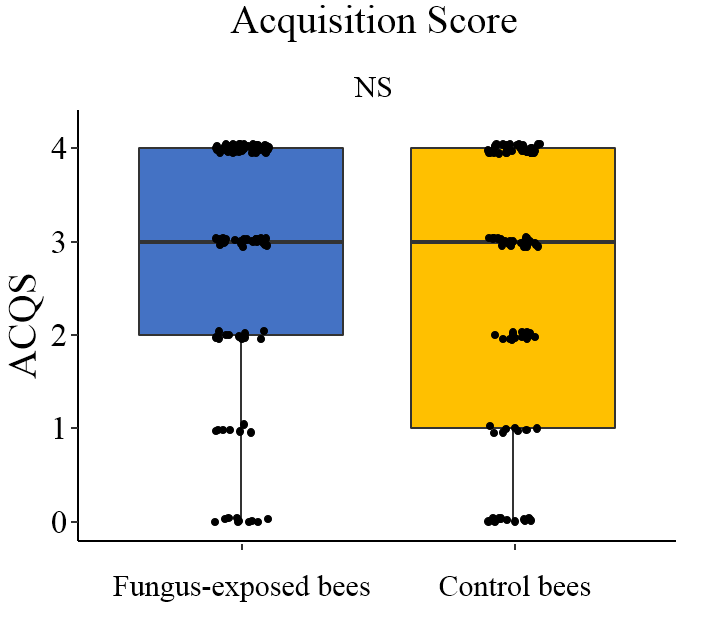
***

**Figure S1: *B. bassiana* did not affect Acquisition Score (ACQS).** Median, quartiles, minimum and maximum Acquisition Scores (ACQS) values of (n = 113) and control (n = 110) bees. Dots represent SRS of individual bees. Individual ACQS was calculated as the number of PER exhibited over the five reinforced trials. Individual ACQS could range from 0 (i.e. bees that never responded to the CS+) to 4 (i.e. bees that responded to the 2^nd^ , the 3^rd^ , the 4^th^ and the 5^th^ CS+ presentation), as bees responding to the 1^st^ CS+ presentation were discarded from the training. At the individual level, fungus-exposed bees did not show higher ACQS than controls (Wilcoxon test, W = 6851, P = 0.17).

**Table SM1: Mortality assay and energetic demand**

Model: lmer (C*onsumption* **~** *treatment* **+** *day* **+** (1**|***cage*))

| Variable | χ^2^ | df | P value |
| --- | --- | --- | --- |
| *Intercept* | 131.16 | 1 | < 2.2e-16 *** |
| *Treatment* | 0.12 | 1 | 0.72 |
| *Day* | 13.7 | 1 | 0.0002 *** |

**Table SM2: Sucrose responsiveness**

Model: glmer (*Response* ~ *Treatment* + *Sucrose-concentration* + (1|*Bee_nr*). Family = binomial (link = "logit")

| Variable | Estimate | Std. Error | Z value | P value |
| --- | --- | --- | --- | --- |
| *Intercept* | -27.23 | 2.64 | -10.30 | <2e-16 *** |
| *Treatment* | -11.10 | 4.35 | -2.55 | 0.011 * |
| *Sucrose concentration* | 165.69 | 18.20 | 9.11 | <2e-16 *** |

**Table SM3: Habituation**

Model: glmer (*Response* ~ *Treatment* + *Trial* + (1 *Bee_nr*)). Family = binomial (link = "logit"). Optimizer = ('bobyqa')

| Variable | χ^2^ | df | P value |
| --- | --- | --- | --- |
| *Intercept* | 75.29 | 1 | < 2.2e-16 *** |
| *Treatment* | 11.53 | 1 | 0.0007*** |
| *Trial* | 544.88 | 1 | < 2.2e-16 *** |

**Table SM4: Associative learning**

Model: glmer (*Response Acquisition* ~ *Treatment* + *Trial* + *CS* + *Treatment*Trial* + *Treatment*CS* + (1|*Bee_nr*). Family = binomial (link = "logit"). Optimizer = ('bobyqa')

| Variable | χ^2^ | df | P value |
| --- | --- | --- | --- |
| *Intercept* | 48.90 | 1 | 2.707e-12 *** |
| *Treatment* | 0.06 | 1 | 0.81 |
| *Trial* | 112.30 | 1 | < 2.2e-16 *** |
| *CS* | 231.83 | 1 | < 2.2e-16 *** |
| *Treatment*Trial* | 2.62 | 1 | 0.11 |
| *Treatment*CS* | 15.79 | 1 | 112e-05 *** |

**Post-hoc test: *lsmeans* (***Treatment*CS , adjust = Dunnett)*

| Contrast | Estimate | Std. Error | df | Z value | P value |
| --- | --- | --- | --- | --- | --- |
| *Bb CS+ vs Water CS+* | 0.368 | 0.268 | NA | 1.370 | 0.54 |
| *Bb CS- vs Water CS-* | -0.766 | 0.319 | NA | -2.395 | 0.081 |
